# Outpatient Embedded Palliative Care for Patients with Advanced Thoracic Malignancy: A Retrospective Cohort Study

**DOI:** 10.3390/curroncol31030105

**Published:** 2024-03-07

**Authors:** Mary C. Boulanger, Margaret D. Krasne, Ethan K. Gough, Samantha Myers, Ilene S. Browner, Josephine L. Feliciano

**Affiliations:** 1 Department of Medical Oncology, Johns Hopkins Sidney Kimmel Comprehensive Cancer Center, Baltimore, MD 21287, USA; 2 Department of Medical Oncology, Dana-Farber Cancer Institute/Massachusetts General Brigham, Boston, MA 02114, USA; 3 Department of Internal Medicine, Johns Hopkins Hospital, Baltimore, MD 21287, USA; 4 Johns Hopkins Bloomberg School of Public Health, Johns Hopkins University, Baltimore, MD 21205, USA; 5 Johns Hopkins Bayview, 301 Lord Mason Drive, Baltimore, MD 21224, USA

**Keywords:** palliative care, thoracic oncology, high-value care

## Abstract

Although cancer care is often contextualized in terms of survival, there are other important cancer care outcomes, such as quality of life and cost of care. The ASCO Value Framework assesses the value of cancer therapies not only in terms of survival but also with consideration of quality of life and financial cost. Early palliative care for patients with advanced cancer is associated with improved quality of life, mood, symptoms, and overall survival for patients, as well as cost savings. While palliative care has been shown to have numerous benefits, the impact of real-world implementation of outpatient embedded palliative care on value-based metrics is not fully understood. We sought to describe the association between outpatient embedded palliative care in a multidisciplinary thoracic oncology clinic and inpatient value-based metrics. We performed a retrospective cohort study of 215 patients being treated for advanced thoracic malignancies with non-curative intent. We evaluated the association between outpatient embedded palliative care and inpatient clinical outcomes including emergency room visits, hospitalizations, intensive care unit admissions, hospital charges, as well as hospital quality metrics including 30-day readmissions, admissions within 30 days of death, inpatient mortality, and inpatient hospital charges. Outpatient embedded palliative care was associated with lower hospital charges per day (USD 3807 vs. USD 4695, *p* = 0.024). Furthermore, patients who received outpatient embedded palliative care had lower hospital admissions within 30 days of death (O.R. 0.45; 95% CI 0.29, 0.68; *p* < 0.001) and a lower inpatient mortality rate (IRR 0.67; 95% CI 0.48, 0.95; *p* = 0.024). Our study further supports that outpatient palliative care is a high-value intervention and alternative models of palliative care, including one embedded into a multidisciplinary thoracic oncology clinic, is associated with improved value-based metrics.

## 1. Introduction

Despite recent advances in treatment, including immunotherapy and targeted therapy, advanced esophageal and lung cancers remain highly morbid and fatal diseases with 5-year survival rates of approximately 5 and 8%, respectively [1,2]. In the United States, lung cancer is the third most common cancer and the leading cause of cancer deaths, with an estimated 127,070 deaths in 2023, or 20.8% of all cancer deaths [3]. Patients with lung cancer may experience numerous symptoms, including fatigue, pain, dyspnea, cough, and insomnia, which are associated with decreased quality of life [4]. Likewise, patients with esophageal cancer may experience fatigue, anxiety, and problems related to eating and have a decreased quality of life [5]. Considering the aggressive nature of these cancers and the associated high symptom burden, it is critical to evaluate and optimize the delivery of cancer care to address patients’ needs, within the context of health systems.

Although cancer care is often contextualized in terms of survival, there are other important cancer care outcomes, such as quality of life and cost of care. One increasingly recognized model that incorporates these outcomes is the American Society of Clinical Oncology (ASCO) Value Framework, which “assesses the value of new cancer therapies based on clinical benefit in terms of life extension or survival” while also taking into consideration “side effects, and improvements in patient symptoms or quality of life in the context of cost” [6]. Value-based frameworks are also increasingly utilized at the system and policy levels for reimbursement and coverage decisions. For example, the Hospital Global Budget program in Maryland was implemented in 2014 with a goal of reducing unnecessary hospital utilization and encouraging primary care to mitigate health care costs and improve clinical outcomes [7].

Palliative care (PC) is an example of a high-value intervention that improves quality of life, mood, and symptoms for patients with advanced cancer and increases the likelihood of discussions of understanding of prognosis, advance care planning, and caregiver needs [8,9,10,11]. Additionally, inpatient PC for patients with serious or terminal illnesses have also been associated with decreased costs of care [12]. Outpatient embedded PC is gaining attention, as it may be a PC model that can mitigate the barriers of additional appointments and time burden for patients [13]. Prior work has demonstrated that the implementation of outpatient embedded PC is feasible and that embedded PC is associated with a reduction in emergency room visits [14,15]. PC teams are multidisciplinary and may involve PC physicians, advanced practice providers, nurses, pharmacists, social workers, psychologists, chaplains, and allied health professionals. The ASCO and the National Comprehensive Cancer Network (NCCN) endorse the integration of PC services within 8 weeks of diagnosis of advanced cancers [16,17]. Although concurrent palliative care is recommended as the standard of care for patients with advanced cancer, it is not universally available or integrated into care, and referrals to palliative care often occur late, if at all [18].

While PC has numerous benefits for patients as described previously, the impact of outpatient embedded PC in a multidisciplinary cancer clinic on inpatient outcomes and hospital value-based metrics for patients with advanced thoracic malignancies has yet to be fully described. Understanding the impact of outpatient embedded PC on hospital value-based metrics could have important implications for hospital resource allocation for PC and thus access to PC for patients. To address this gap in understanding, we conducted a retrospective cohort study to evaluate real-world implementation of outpatient embedded PC and the association with inpatient health care utilization outcomes. We sought to evaluate associations between outpatient embedded palliative care and inpatient outcomes including emergency room visits, hospitalizations, intensive care unit admissions, 30-day readmissions, admissions within 30 days of death, inpatient mortality, time in hospice, and hospital charges. The goal of our study was to build on prior work examining the implementation of outpatient embedded PC to provide novel insight into the real-world implementation of outpatient embedded PC and health care utilization.

## 2. Materials and Methods

### 2.1. Study Population

**Figure 1:**
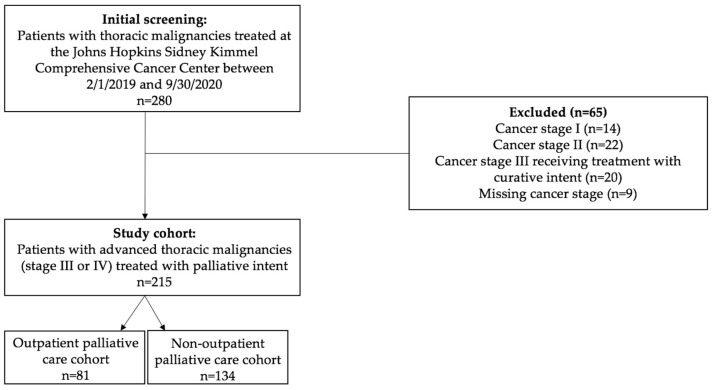
Cohort selection diagram.

We performed a retrospective cohort study (IRB #00275889) of adult patients (≥18 years old) with advanced thoracic malignancies (stage III or IV cancer) treated with palliative intent at the Johns Hopkins Sidney Kimmel Comprehensive Cancer Center between 1 February 2019 and 30 September 2020. We identified patients from the oncology and palliative care clinicians’ patient panel. Exclusion criteria included patients with stage I or II cancer, stage III cancer receiving treatment with curative intent, and those missing cancer staging (Figure 1).

During the study time period, a PC clinician (physician or advanced practice provider) was embedded within the thoracic oncology multidisciplinary clinic at Johns Hopkins Bayview alongside medical oncology, radiation oncology, thoracic surgery, and interventional pulmonology clinicians during three days of a five-day clinic week. The thoracic oncology clinic has a triage nurse, social worker, and pastoral care for multidisciplinary comprehensive care. Patients were referred to PC at the discretion of their primary thoracic medical oncologist or radiation oncologist, with the goal of patients being seen by the PC clinician on the same day or within 7 days of referral, either in person or by a telehealth visit. Nineteen patients in our cohort were enrolled in a clinical trial for early PC in patients with advanced lung cancer treated with palliative intent, and these patients were enrolled within 12 weeks of diagnosis of advanced disease. Prior to our study time period, which encompasses the opening of the embedded PC clinic, patients were referred to a free-standing PC clinic at Johns Hopkins Hospital. The primary oncology team maintained their role as the primary prescriber for patients with guidance from the PC clinician.

### 2.2. Data Collection

We collected clinical data via chart reviews through the electronic health record system called Epic. The sociodemographic and clinical variables included age, sex, race, insurance type, marital status, zip code, cancer type, cancer stage at diagnosis, dates of first outpatient oncology and PC visits, and date and location of death. The receipt of PC was defined as at least one outpatient PC visit documented in Epic. We estimated the median income by zip code as reported by the United States Census Bureau [19]. We identified 9 high-risk zip codes (Supplemental Figure S1) in East Baltimore that have previously been determined by our institutional cancer registry to have elevated rates of cancer mortality compared to other zip codes in Baltimore (IRB #00160610). We collected clinical data regarding emergency room visits, hospitalizations, and ICU admissions from the Johns Hopkins Medicine hospitals (Johns Hopkins Hospital, Johns Hopkins Bayview Medical Center, Sibley Memorial Hospital, Suburban Hospital, and Howard County General Hospital).

### 2.3. Statistical Analyses

We used Fisher’s exact test and Student’s t-test to determine whether the sociodemographic and clinical variables differed based on outpatient PC status for categorical and continuous variables, respectively. Hospital utilization and charge data were obtained from the Johns Hopkins Medicine Casemix/Datamart database, which is created and used for mandatory reporting to the state of Maryland and includes casemix information and billing data. The hospital charges included all charges regulated by the Health Service Cost Review Commission (HSCRC), Maryland’s hospital rate-setting authority. These data were used to calculate the average charge per day. Hospital charges from Sibley Memorial Hospital, Suburban Hospital, and Howard County General Hospital were not available, thus these hospitals were excluded from the charge analysis.

We compared the clinical outcomes for those who received outpatient PC and those who did not, including the number of emergency room visits, number of hospitalizations, number of ICU admissions, average and total length of stay, average hospital charges per admission and per day, total hospital charges, 30-day readmission rates, admissions within 30 days of death, inpatient mortality, average days receiving hospice services, and likelihood of PC consults during any inpatient admission. Incident rate ratios, average differences, and odds ratios were used to estimate differences between clinical outcomes for event counts, continuous outcomes, and binary outcomes, respectively. Effect sizes and *p*-values for event counts were calculated using generalized linear regression with a Poisson distribution, a log link, and person-time of observation as an offset. The person-time contribution [20,21] of each patient was defined as the time from the start of the study period (1 February 2019) until death or the end of the study period (30 September 2020), whichever came first. Simple linear regression was used to calculate effect sizes and *p*-values for continuous outcomes, and logistic regression was used for binary outcomes. Additionally, we created multivariable models to evaluate independent associations between outpatient PC and clinical outcomes. Whether a patient received outpatient embedded PC or not was the primary exposure of interest. Covariates were included in each model based on prior clinical knowledge and a priori association of these variables with our primary exposure and outcomes of interest. The missing data were minimal (see Table 1 and Table 2). Complete case analyses were used to deal with missing values. Several of the covariates were also used in the multivariable models. All analyses were conducted in R version 4.2.0.

## 3. Results

The analyzed cohort included 215 patients being treated for advanced thoracic malignancies with non-curative intent (Figure 1). The study cohort’s sociodemographic characteristics are summarized in Table 1. The cohort’s median age was 66 years, with 52% female patients, 69% White patients, and 60% married patients. Approximately 74% of the cohort had non-small cell lung cancer (NSCLC) and 82% had stage IV disease at the time of diagnosis. Thirty-eight percent (81/215) of the cohort received outpatient palliative care (PC). A higher proportion of males (59% vs. 41%, *p* = 0.011), White patients (77% vs. 64%, *p* = 0.047), and Maryland residents (90% vs. 79%, *p* = 0.039) received PC than not. There were no significant differences in insurance type, marital status, high-risk zip code, cancer type, or stage at diagnosis between the outpatient PC (OPC) and non-OPC cohorts. For the entire cohort, 20% of patients died within 90 days from their first oncology visit at Johns Hopkins, 15% died within 90–180 days, and 65% lived beyond 180 days.

The clinical outcomes for those who did and did not receive outpatient embedded PC are summarized in Table 2. Those who received outpatient embedded PC had lower hospital charges per day (USD 3807 vs. USD 4695; *p* = 0.024) compared to patients who did not receive OPC. The patients in the outpatient embedded PC cohort were less likely to have a hospital admission within 30 days of death (OR 0.56, 95% CI 0.36, 0.88; *p* = 0.011) compared to the non-OPC cohort. The inpatient mortality rate (IRR 0.62, 95% CI 0.44, 0.87; *p* = 0.006) and inpatient mortality risk (OR 0.58, 95% CI 0.39, 0.87; *p* = 0.009) was lower in the OPC cohort compared to the non-OPC cohort. We did not identify significant differences between the OPC versus non-OPC cohorts in the number of ER visits, hospitalizations, ICU admissions, average or total length of stay, average hospital charges per admission, total hospital charges, readmission within 30 days from discharge, average days receiving hospice, or likelihood of inpatient palliative care consultation during any inpatient admission.

We performed multivariable analyses to identify independent associations between outpatient PC exposure and clinical outcomes including emergency room use, hospitalizations, ICU admissions, readmissions within 30 days of discharge, hospitalization within 30 days of death, and inpatient mortality (Table 3). In the multivariable analyses, outpatient PC was independently associated with lower odds of hospital admission within 30 days of death (O.R. 0.45; 95% CI 0.29, 0.68; *p* < 0.001) and a lower inpatient mortality rate (IRR 0.67; 95% CI 0.48, 0.95; *p* = 0.024). We did not identify an association between outpatient PC and the odds of emergency room visits, hospitalizations, ICU admissions, re-admissions within 30 days of a previous hospital discharge, or time in hospice.

## 4. Discussion

Our study describes how an outpatient embedded PC model in a thoracic oncology multidisciplinary clinic for patients with advanced thoracic malignancies is associated hospital value-based metrics, including lower inpatient mortality rate, decreased likelihood of hospital admission within 30 days of death, and lower hospital charges per day. Our observations further support outpatient embedded palliative care as a high-value practice.

Both inpatient and outpatient models of palliative care have demonstrated improved quality metrics, such as inpatient mortality and hospital resource utilization. Brumley et al. performed a randomized trial of patients with terminal illnesses, including chronic obstructive pulmonary disease, congestive heart failure, and cancer, with a life expectancy of less than one year [22]. The patients enrolled in in-home PC were more likely to die at home (*p* < 0.001), less likely to visit the ER (*p* = 0.01), and less likely to be admitted (*p* < 0.001), with lower inpatient hospital costs within the last 30 days of life [22]. Furthermore, Vranas et al. retrospectively evaluated 23,142 patients with advanced NSCLC in the Veterans Affairs HealthCare System who received inpatient or outpatient PC. Outpatient PC was associated with a reduced hospitalization rate within 30 days of death (aIRR, 0.64; 95% CI, 0.59–0.70) and lower likelihood of ER visits (aIRR 0.86; 95% CI 0.77–0.96) [23].

Additionally, various models of palliative care are associated with lower costs. A randomized controlled trial by Gade, et al. demonstrated that patients with a life expectancy of one year who were randomized to inpatient PC versus standard of care had a 6-month net cost reduction of USD 4855 per patient (*p* = 0.001) [24]. Another study by Morrison, et al. analyzed administrative data from 2278 patients who received inpatient PC matched to patients who received usual care and showed lower costs per admission and costs per day for patients who received inpatient PC [12]. We identified a significant difference in daily hospital charges for our OPC cohort but did not observe a difference in average or total hospital charges. As more patients in the non-OPC arm lived out of state (20.9% vs. 9.9%, Table 1) and because we were unable to capture outcomes outside of our health system, we suspect that we were disproportionately unable to capture ER visits, hospitalizations, ICU admissions, and hospital charges for non-OPC patients. Despite the likelihood of missing hospital and charge data for out of state patients, more of whom were in the non-OPC arm, we still observed reduced charges per day in our OPC arm. Furthermore, our differences in findings may be attributed partially to our collection of charge and hospitalization data throughout the patient’s disease course in this time period, not just the last 30 days or solely in patients with a life expectancy of less than one year.

While most NCI-designated cancer centers utilize free-standing PC clinics, there is growing interest in embedded PC clinics. Investigators at The Ohio State University reported in a retrospective cross-sectional cohort study that patients seen in a 12-month time period when PC was embedded, versus a 12-month period when patients were referred to a free-standing PC clinic, had a reduction in ER visits (adjusted RR 0.74; 95% CI 0.58–0.94) [15]. Similarly to these investigators, we were only able to capture outcomes that occurred within our health system. However, we were able to capture a longer follow-up period than 12 months. Furthermore, our subset of patients who received OPC was larger than was reported by Gast et al. and did not include patients with curative-intent disease, perhaps selecting for a patient population who would benefit more from PC.

Though we could not assess for this in our study, we speculate that outpatient embedded PC may have impacted inpatient resource utilization measures due to multiple factors. Patients who received outpatient PC may have had a different philosophy towards their illness at baseline. Furthermore, patients who received outpatient PC may have had more discussions about their understanding of their disease, prognosis, care planning, and wishes for end-of-life care. It is also possible that the presence of embedded outpatient PC may have off-target effects on oncology clinicians and patients due increased PC awareness, education, and support. Our data further support an embedded model in the outpatient setting as being associated with improved inpatient value-based metrics, which should strongly be considered when hospitals discuss resource allocation for PC.

Our study has several limitations to consider. We have a small sample size which may limit our ability to detect differences in outcomes and our study is retrospective. Retrospective studies are inherently limited as they cannot determine causality and depend on the completeness and accuracy of documentation in the electronic medical record. Our study window includes the beginning of the COVID-19 pandemic and thus may reflect decreased access to appointments, emergency room visits, and hospitalizations. Our study was retrospective and non-randomized and thus relied on the discretion of the primary oncology team to refer to PC, which may introduce inherent bias in the patient population and impact outcomes of measures, as it is possible that patients with higher symptom burdens or anticipated shorter survival were referred to PC. Furthermore, our analysis did not stratify the OPC cohort by early versus late OPC, which may also impact outcomes. Additionally, we did not assess the number or frequency of visits with palliative care or ensure that the TEAM (Time, Education, Assessment, Management) approach based on previous randomized trials was used, which may also impact outcomes [25,26]. While the majority of patients were seen by our embedded PC team, there were a limited number of patients who were seen by a free-standing PC clinic during the same time frame and were included in our analysis. Furthermore, we did not assess patients who were referred to outpatient PC and did not subsequently establish care with PC. As hospital charge data were not available for Sibley Memorial Hospital, Suburban Hospital, and Howard County General Hospital, this introduces the possibility of systematic bias and future studies should investigate how to attain more broad charge data. Finally, our study was focused on patients with thoracic malignancies, and therefore may not be generalizable to all cancer types.

Despite the various limitations of our study, there are notable strengths to this analysis. First, this study may represent a more real-world experience of how embedded palliative care is implemented in a clinic, and even by telehealth. Secondly, our study attempted to capture outcomes throughout the continuum of care for patients with advanced thoracic cancer and still observed improved value-based metrics associated with PC. Finally, our analysis describes hospital charges in a state where hospital reimbursements are capped under the Maryland Global Budget Revenue. High-value interventions such as PC may be particularly relevant for states that are considering this type of reimbursement model in the future. Outpatient embedded PC may be a care model that can improve access to PC by reducing known barriers to PC, including time and logistical and organizational barriers [13,27]. Exploring care models that may improve PC access is particularly relevant for patients with advanced thoracic malignancies as they experience a high symptom burden and decreased quality of life.

## 5. Conclusions

Value-based frameworks are increasingly considered in individual-, system-, and policy-level decisions in cancer care. While the ASCO and the NCCN practice guidelines endorse the early integration of PC for patients with advanced cancer, there are often barriers to providing this standard of care treatment such as availability of PC clinicians and funding challenges for PC services [16,17,28,29,30]. The real-world implementation of outpatient embedded PC appears to be a high-value practice associated with improved hospital value-based metrics. Outpatient embedded PC, versus a free-standing PC clinic, may also be more patient-centric if aligned with oncology or infusion visits to reduce the burden of time, transportation, and associated costs to the patients. Future studies are needed to understand the impacts of outpatient embedded palliative care on value-based metrics for health care systems and, most importantly, on patient-reported outcomes. Likewise, value-based metrics, such as inpatient mortality, also require further investigation as to whether they lead to patient-centric goal concordant care.

## Figures and Tables

**Table 1:** Study population demographics and disease characteristics.

	Total (*n* = 215)	OPC (*n* = 81)	Non-OPC (*n* = 134)	*p*-Value
**Age (median, IQR)**	66.17 (59.82, 75.12)	65.78 (59.20, 74.08)	66.40 (60.15, 75.69)	0.285
**Sex (*n* (%))**				
Female	112 (52.1%)	33 (40.7%)	79 (59.0%)	0.011 *
Male	103 (47.9%)	48 (59.3%)	55 (41.0%)	
**Race (*n* (%))**				
White	148 (68.8%)	62 (76.5%)	86 (64.2%)	0.047 *
Black or African American	46 (21.4%)	9 (11.1%)	37 (27.6%)	0.006 *
Asian	8 (3.7%)	3 (3.7%)	5 (3.7%)	1.000
Other	12 (5.6%)	6 (7.4%)	6 (4.5%)	0.371
Missing	1 (0.5%)	1 (1.2%)	0 (0%)	
**Insurance (*n* (%))**				
Medicare	34 (15.3%)	11 (13.6%)	23 (17.2%)	0.565
Medicare with supplemental insurance	107 (49.8%)	40 (49.4%)	67 (50.0%)	1.000
Medicaid/No insurance	22 (10.2%)	6 (7.4%)	16 (11.9%)	0.188
Private/Self pay	52 (24.2%)	24 (29.6%)	28 (20.9%)	0.238
**Marital status (*n* (%))**				
Single	36 (16.7%)	9 (11.1%)	27 (20.1%)	0.130
Married	129 (60.0%)	53 (65.4%)	76 (56.7%)	0.195
Separated, Divorced, Widowed, or Unknown	49 (22.8%)	18 (22.2%)	31 (23.1%)	1.000
Missing	1 (0.5%)	1 (1.2%)	0 (0%)	
**High risk zip code (*n* (%))**				
No	188 (87.4%)	74 (91.4%)	114 (85.1%)	0.207
Yes	27 (12.6%)	7 (8.6%)	20 (14.9%)	
**Median income by zip code (*n* (%))**				
<USD 40,000	10 (4.7%)	4 (4.9%)	6 (4.5%)	1.000
USD 40,000–100,000	139 (64.7%)	48 (59.3%)	91 (67.9%)	0.139
>USD 100,000	63 (29.3%)	29 (35.8%)	34 (25.4%)	0.164
Missing	3 (1.4%)	0 (0%)	3 (2.2%)	
**State of residence (*n* (%))**				
Maryland	179 (83.3%)	73 (90.1%)	106 (79.1%)	0.039 *
Other	36 (16.7%)	8 (9.9%)	28 (20.9%)	
**Cancer type (*n* (%))**				
NSCLC	159 (74.0%)	63 (77.8%)	96 (71.6%)	0.253
SCLC	18 (8.4%)	4 (4.9%)	14 (10.4%)	0.208
Esophageal	21 (9.8%)	8 (9.9%)	13 (9.7%)	1.000
Other	14 (6.5%)	4 (4.9%)	10 (7.5%)	0.577
Missing	3 (1.4%)	2 (2.5%)	1 (0.7%)	
**Cancer stage at diagnosis (*n* (%))**				
III	38 (17.7%)	12 (14.8%)	26 (19.4%)	0.463
IV	177 (82.3%)	69 (85.2%)	108 (80.6%)	
**Time from first outpatient oncology visit to date of death (*n* (%))**				
<90 days	43 (20.0%)	7 (8.6%)	36 (26.9%)	0.001 *
90–180 days	32 (14.9%)	9 (11.1%)	23 (17.2%)	0.242
>180 days	139 (64.7%)	65 (80.2%)	74 (55.2%)	<0.001 *
Missing	1 (0.5%)	0 (0%)	1 (0.7%)	

OPC = outpatient palliative care. * Statistically significant (*p* < 0.05).

**Table 2:** Outpatient embedded palliative care and clinical outcomes, N = 215.

	OPC (*n* = 81)	Non-OPC (*n* = 134)	Effect Size (95%CI)	*p*-Value
Number of ER visits (n/person-years of follow-up)	1.93 (154/79.8)	1.47 (191/130.3)	1.32 (0.89, 1.96) ^1^	0.173
Number of hospitalizations (n/person-years of follow-up)	1.92 (153/79.8)	1.74 (227/130.3)	1.10 (0.91, 1.34) ^1^	0.329
Number of ICU admissions (n/person-years of follow-up)	0.31 (25/79.8)	0.32 (42/130.3)	0.97 (0.66, 1.43) ^1^	0.886
Average length of stay (mean (sd))	6.63 (5.61)	6.84 (6.76)	−0.20 (−2.69, 2.28) ^2^	0.872
Missing (*n*)	1	2		
Total length of stay (mean (sd))	11.58 (14.63)	10.75 (14.00)	0.83 (−2.64, 4.30) ^2^	0.638
Average hospital charge per admission at Johns Hopkins Hospital or Johns Hopkins Bayview (mean (sd))	24,887 (22,325)	29,191 (34,565)	−4304 (−15,419, 6811) ^2^	0.448
Missing (*n*)	1	2		
Average hospital charge per day at Johns Hopkins Hospital or Johns Hopkins Bayview (mean (sd))	3807 (1465)	4695 (3693)	−887 (−1657, −118) ^2^	0.024 *
Missing (*n*)	2	3		
Total hospital charges (mean (sd))	42,579 (56,354)	43,578 (65,811)	−999 (−20,465, 18,467) ^2^	0.920
Missing (*n*)	1	2		
Readmission within 30 days from prior discharge (n/person-years of follow-up)	0.59 (47/79.8)	0.59 (77/130.3)	1.00 (0.74, 1.35) ^1^	0.984
Hospital admission within 30 days of death (n/N)	0.45 (25/56)	0.59 (56/95)	0.56 (0.36, 0.88) ^3^	0.011 *
Missing (*n*)	1	6		
Inpatient mortality rate (n/person-years of follow-up)	0.19 (14/74.8)	0.30 (33/109.0)	0.62 (0.44, 0.87) ^1^	0.006 *
Missing (*n*)	3	13		
Inpatient mortality risk (n/N)	0.18 (14/78)	0.27 (33/121)	0.58 (0.39, 0.87) ^3^	0.009 *
Missing (*n*)	3	13		
Average number of days in hospice for those enrolled in hospice (mean (sd))	14.7 (20.7)	13.9 (31.0)	0.87 (−6.49, 8.22) ^2^	0.818
Missing (*n*)	1	1		
Received inpatient palliative care consult during any inpatient admission (n/N)	0.40 (32/81)	0.31 (41/134)	1.48 (0.68, 3.22) ^3^	0.321

^1^ Incident rate ratio. ^2^ Average difference. ^3^ Odds ratio. * Statistically significant (*p* < 0.05).

**Table 3:** Multivariable analysis of outpatient embedded palliative care and clinical outcomes.

	Emergency Room Visits	Hospitalizations	ICU Admissions	Readmission within 30 Days	Hospital Admission within 30 Days of Death	Inpatient Mortality (Rate)	Inpatient Mortality (Odds)	Time in Hospice < 7 Days	Time in Hospice 8–14 Days	Time in Hospice > 14 Days
	IRR(95%CI); *p*-Value	IRR(95%CI); *p*-Value	IRR(95%CI); *p*-Value	IRR(95%CI); *p*-Value	OR(95%CI); *p*-Value	IRR(95%CI; *p*-Value	OR(95%CI); *p*-Value	OR(95%CI); *p*-Value	OR(95%CI); *p*-Value	OR(95%CI); *p*-Value
Outpatient PC vs. no outpatient PC	1.45 (1.00, 2.10); 0.050	1.16 (0.86, 1.58); 0.336	1.03 (0.69, 1.54); 0.889	0.92 (0.54, 1.59); 0.775	0.45 (0.29, 0.68); <0.001 *	0.67 (0.48, 0.95); 0.024 *	0.65 (0.36, 1.18); 0.158	0.64 (0.36, 1.12); 0.120	1.20 (0.60, 2.39); 0.609	1.52 (0.70, 3.28); 0.291

* Statistically significant (*p* < 0.05) Note: Multivariable model included the following covariates: age, sex (female, male), race (White, Black or African American, Asian, other), insurance (Medicare, Medicare + supplemental, Medicaid/none, private/self), marital status (single, married, separated/divorced/widowed/unknown), high risk zip code (yes, no), median income by zip code (<USD 40,000, USD 40,000–100,000, >USD 100,000), state (Maryland, other), cancer type (NSCLC, SCLC, esophageal, other), and cancer stage (III, IV).

## Data Availability

The data presented in this study are available on request from the corresponding author.

## Supplementary Materials

The following supporting information can be downloaded at: https://www.mdpi.com/article/10.3390/curroncol31030105/s1, Figure S1: High-risk zip codes in East Baltimore.

## References

[1] Survelliance, Epidemiology, and End Results Program: Esophagus. https://seer.cancer.gov/statistics-network/explorer/application.html?site=17&data_type=4&graph_type=6&compareBy=sex&chk_sex_1=1&race=1&age_range=1&stage=106&advopt_precision=1&advopt_show_ci=on&hdn_view=0&advopt_show_apc=on&advopt_display=2.

[2] Survelliance, Epidemiology, and End Results Program: Lung and Bronchus. https://seer.cancer.gov/statistics-network/explorer/application.html?site=47&data_type=4&graph_type=5&compareBy=stage&chk_stage_106=106&series=age_range&chk_age_range_1=1&sex=1&race=1&advopt_precision=1&advopt_show_ci=on&hdn_view=0&advopt_show_apc=on&advopt_display=2.

[3] Survelliance, Epidemiology, and End Results Program: Cancer Stat Facts: Lung and Bronchus Cancer. https://seer.cancer.gov/statfacts/html/lungb.html.

[4] Hechtner M., Eichler M., Wehler B., Buhl R., Sebastian M., Stratmann J., Schmidberger H., Gohrbandt B., Peuser J., Kortsik C. (2019). Quality of life in NSCLC survivors—A multicenter cross-sectional study. J. Thorac. Oncol..

[5] Dalhammar K., Kristensson J., Falkenback D., Rasmussen B.H., Malmström M. (2022). Symptoms, problems and quality of life in patients newly diagnosed with oesophageal and gastric cancer—A comparative study of treatment strategy. BMC Cancer.

[6] Schnipper L.E., Davidson N.E., Wollins D.S., Blayney D.W., Dicker A.P., Ganz P.A., Hoverman J.R., Langdon R., Lyman G.H., Meropol N.J. (2016). Updating the American Society of Clinical Oncology Value Framework: Revisions and Reflections in Response to Comments Received. J. Clin. Oncol..

[7] Roberts E.T., McWilliams J.M., Hatfield L.A., Gerovich S., Chernew M.E., Gilstrap L.G., Mehrotra A. (2018). Changes in Health Care Use Associated With the Introduction of Hospital Global Budgets in Maryland. JAMA Intern. Med..

[8] Palliative Care in Cancer. https://www.cancer.gov/about-cancer/advanced-cancer/care-choices/palliative-care-fact-sheet.

[9] Thomas T.H., Jackson V.A., Carlson H., Rinaldi S., Sousa A., Hansen A., Kamdar M., Jacobsen J., Park E.R., Pirl W.F. (2019). Communication Differences between Oncologists and Palliative Care Clinicians: A Qualitative Analysis of Early, Integrated Palliative Care in Patients with Advanced Cancer. J. Palliat. Med..

[10] Temel J.S., Greer J.A., Muzikansky A., Gallagher E.R., Admane S., Jackson V.A., Dahlin C.M., Blinderman C.D., Jacobsen J., Pirl W.F. (2010). Early palliative care for patients with metastatic non-small-cell lung cancer. N. Engl. J. Med..

[11] Greer J.A., Jackson V.A., Meier D.E., Temel J.S. (2013). Early integration of palliative care services with standard oncology care for patients with advanced cancer. CA Cancer J. Clin..

[12] Morrison R.S., Penrod J.D., Cassel J.B., Caust-Ellenbogen M., Litke A., Spragens L., Meier D.E. (2008). Palliative Care Leadership Centers’ Outcomes Group. Cost savings associated with US hospital palliative care consultation programs. Arch. Intern. Med..

[13] Agne J.L., Bertino E.M., Grogan M., Benedict J., Janse S., Naughton M., Eastep C., Callahan M., Presley C.J. (2021). Too Many Appointments: Assessing Provider and Nursing Perception of Barriers to Referral for Outpatient Palliative Care. Palliat. Med. Rep..

[14] Bertino E.M., Grogan M.M., Benedict J.A., Agne J.L., Janse S., Eastep C., Sullivan D., Gast K.C., Naughton M.J., Presley C.J. (2023). Feasibility of an embedded palliative care clinic model for patients with an advanced thoracic malignancy. Support Care Cancer.

[15] Gast K.C., Benedict J.A., Grogan M., Janse S., Saphire M., Kumar P., Bertino E.M., Agne J.L., Presley C.J. (2022). Impact of an Embedded Palliative Care Clinic on Healthcare Utilization for Patients With a New Thoracic Malignancy. Front. Oncol..

[16] Ferrell B.R., Temel J.S., Temin S., Alesi E.R., Balboni T.A., Basch E.M., Firn J.I., Paice J.A., Peppercorn J.M., Phillips T. (2017). Integration of Palliative Care Into Standard Oncology Care: American Society of Clinical Oncology Clinical Practice Guideline Update. J. Clin. Oncol..

[17] Dans M., Smith T., Back A., Baker J.N., Bauman J.R., Beck A.C., Block S., Campbell T., Case A.A., Dalal S. (2017). NCCN Guidelines Insights: Palliative Care, Version 2.2017. J. Natl. Compr. Canc. Netw..

[18] Smith C.B., Phillips T., Smith T.J. (2017). Using the New ASCO Clinical Practice Guideline for Palliative Care Concurrent with Oncology Care Using the TEAM Approach. Am. Soc. Clin. Oncol. Educ. Book.

[19] Census Bureau U.S. Explore Census Data. https://data.census.gov/cedsci/table?g=0500000US48301&tid=ACSST5Y2019.S1901.

[20] Greenland S., Modern Epidemiology K.G., Rothman S., Lash T.L. (2008). Introduction to Regression Models.

[21] Szcklo M.N., Nieto F.J. (2014). Epidemiology: Beyond the Basics.

[22] Brumley R., Enguidanos S., Jamison P., Seitz R., Morgenstern N., Saito S., McIlwane J., Hillary K., Gonzalez J. (2007). Increased satisfaction with care and lower costs: Results of a randomized trial of in-home palliative care. J. Am. Geriatr. Soc..

[23] Vranas K.C., Lapidus J.A., Ganzini L., Slatore C.G., Sullivan D.R. (2020). Association of Palliative Care Use and Setting With Health-care Utilization and Quality of Care at the End of Life Among Patients With Advanced Lung Cancer. Chest.

[24] Gade G., Venohr I., Conner D., McGrady K., Beane J., Richardson R.H., Williams M.P., Liberson M., Blum M., Della Penna R. (2008). Impact of an inpatient palliative care team: A randomized control trial. J. Palliat. Med..

[25] Sedhom R., Gupta A., MacNabb L., Smith T.J. (2020). The Impact of Palliative Care Dose Intensity on Outcomes for Patients with Cancer. Oncologist.

[26] Bakitas M.A., El-Jawahri A., Farquhar M., Ferrell B., Grudzen C., Higginson I., Temel J.S., Zimmermann C., Smith T.J. (2017). The TEAM Approach to Improving Oncology Outcomes by Incorporating Palliative Care in Practice. J. Oncol. Pract..

[27] Parajuli J., Hupcey J.E. (2021). A Systematic Review on Barriers to Palliative Care in Oncology. Am. J. Hosp. Palliat. Care.

[28] Schnipper L.E., Smith T.J., Raghavan D., Blayney D.W., Ganz P.A., Mulvey T.M., Wollins D.S. (2012). American Society of Clinical Oncology identifies five key opportunities to improve care and reduce costs: The top five list for oncology. J. Clin. Oncol..

[29] Aldridge M.D., Hasselaar J., Garralda E., van der Eerden M., Stevenson D., McKendrick K., Centeno C., Meier D.E. (2016). Education, implementation, and policy barriers to greater integration of palliative care: A literature review. Palliat. Med..

[30] Sedhom R., Kamal A.H. (2022). Is Improving the Penetration Rate of Palliative Care the Right Measure?. JCO Oncol. Pract..

