# Supplementary material for: Outpatient Embedded Palliative Care for Patients with Advanced Thoracic Malignancy: A Retrospective Cohort Study
Source: Curr Oncol. 2024 Mar 7;31(3):1389–99. doi: 10.3390/curroncol31030105 (PMC10968799; doi:10.3390/curroncol31030105)
Supplement: Supplementary file 1 [file curroncol-31-00105-s001.zip › curroncol-2776057-supplementary.pptx]

## Slide 1
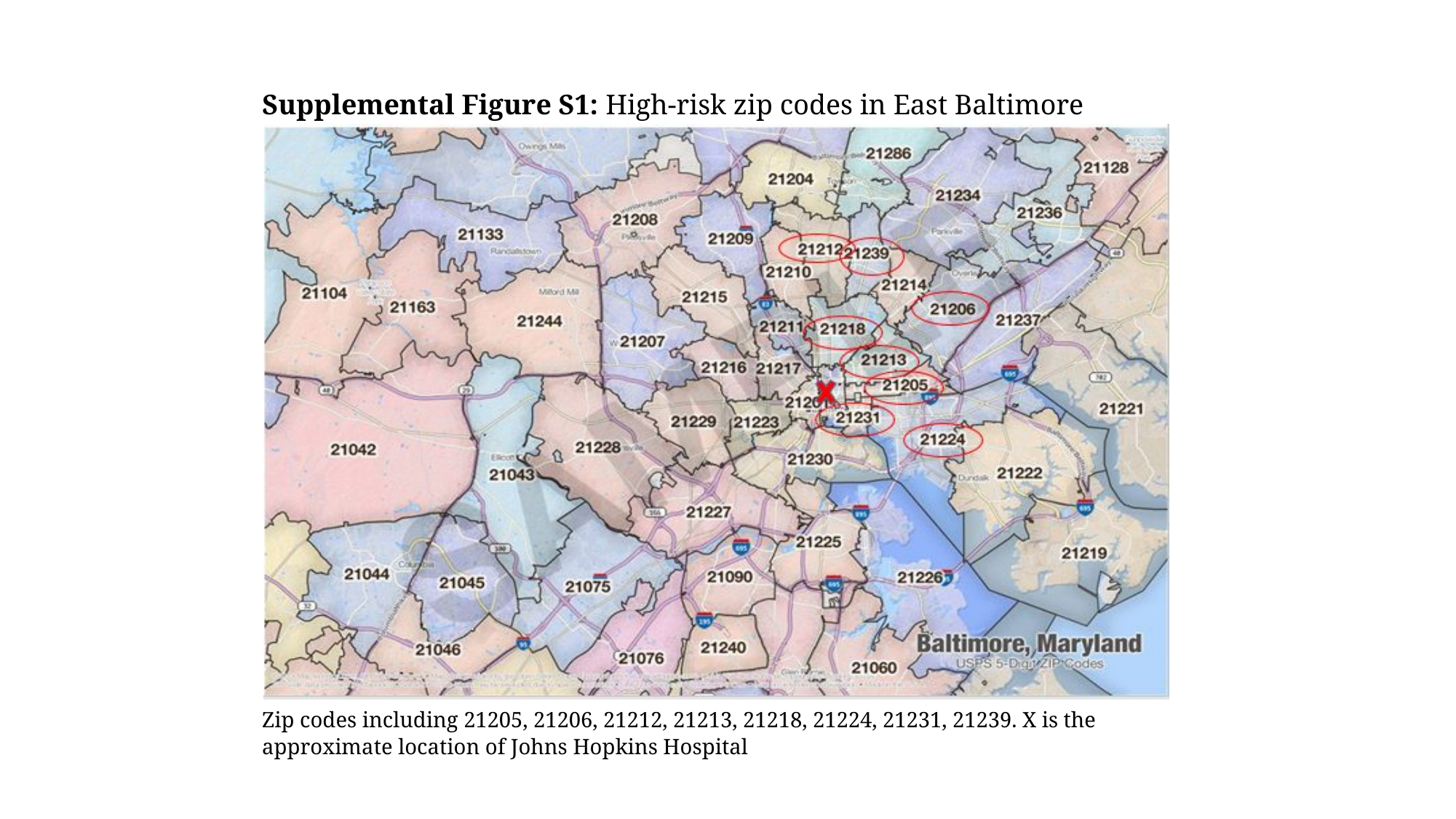

Supplemental Figure S1: High-risk zip codes in East Baltimore
Zip codes including 21205, 21206, 21212, 21213, 21218, 21224, 21231, 21239. X is the approximate location of Johns Hopkins Hospital
